# Label‐Free Microfluidic Apheresis of Circulating Tumor Cell Clusters

**DOI:** 10.1002/advs.202405853

**Published:** 2024-08-28

**Authors:** Li Zhan, Jon Edd, Avanish Mishra, Mehmet Toner

**Affiliations:** ^1^ Center for Engineering in Medicine and Surgery Massachusetts General Hospital Boston MA 02129 USA; ^2^ Harvard Medical School Boston MA 02115 USA; ^3^ Cancer Center Massachusetts General Hospital Boston MA 12129 USA; ^4^ Shriners Hospitals for Children Boston MA 02114 USA

**Keywords:** apheresis, circulating tumor cell clusters, label‐free sorting, microfluidic

## Abstract

Screening liters of blood (i.e., apheresis) represents a generalized approach to promote the reliable access to circulating tumor cell clusters (CTCCs), which are known to be highly metastasis‐competent, yet ultrarare. However, no existing CTCC sorting technology has demonstrated high throughput, high yield, low shear stress, and minimal blood dilution simultaneously as required in apheresis. Here, a label‐free method is introduced termed Precision Apheresis for Non‐invasive Debulking of cell Aggregates (PANDA) to continuously isolate CTCCs from undiluted blood to clean buffer through size sorting, processing 1.4 billion cells per second. The cell focusing is optimized within whole blood leveraging secondary transverse flow and margination. The PANDA chip recovers >90% of spiked ≈24 rare HeLa cell clusters from 100 mL undiluted blood samples (equivalent to ≈500 billion blood cells) at 1 L h^−1^ throughput, with ≤20s device residence time, ≤15 Pa shear stress, and >99.9% return of blood components. The technology lays the groundwork for future routine isolation to increase the recovery of these ultrarare yet clinically significant tumor cell populations from large volumes of blood to advance cancer research, early detection, and treatment.

## Graphical abstract

A label‐free method is reported termed Precision Apheresis for Non‐invasive Debulking of cell Aggregates (PANDA) to continuously isolate circulating tumor cell clusters (CTCCs) from undiluted blood to clean buffer through size sorting at 1L h^−1^ throughput, processing 1.4 billion cells per second. The technology lays the groundwork to increase the recovery of CTCCs from large volumes of blood to advance cancer research, early detection, and treatment.

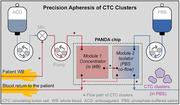

## 1 Introduction

Circulating tumor cells (CTCs), originated from sites throughout the body, can seed metastasis by traveling through the bloodstream,^[^ ^1^ ^]^ providing a non‐invasive liquid biopsy for sampling of cancer. Notably, CTC clusters (CTCCs) arise from groups of tumor cells that together enter the circulation,^[^ ^2^, ^3^ ^]^ though attached leukocytes are also observed.^[^ ^4^ ^]^ Despite being even rarer than single CTCs,^[^ ^5^, ^6^, ^7^ ^]^ CTCCs, on the order of 1 in 10 mL of blood containing ≈50 billion blood cells, were found to be more metastatic than single CTCs.^[^ ^3^ ^]^ Furthermore, their presence is associated with much poorer clinical outcomes.^[^ ^4^, ^6^, ^8^, ^9^, ^10^ ^]^ Indeed, the research efforts to understand and utilize these ultrarare yet clinically significant cell populations are severely hindered by the limited volume of blood (i.e., 10–20 mL) used for analysis. Approaches to process liters of blood are needed to increase recovery of CTCCs in broad ranges of patient populations. Such approaches will permit important applications including early CTCC‐based cancer diagnostics where abundance of CTCCs is extremely low, and ex vivo CTCCs culture/characterizations to assist cancer treatment for personalized oncology.

Apheresis is a routine clinical procedure wherein blood is drawn, continuously centrifuged to extract desired products, and subsequently returned to the circulation. For instance, leukapheresis interrogates the entire blood volume (≈5 L in adults) at 3 L h^−1^ to isolate mononuclear cells, which form the buffy coat layer atop the red blood cell (RBC) layer after bulk centrifugation.^[^ ^11^, ^12^, ^13^ ^]^ Leukapheresis products have recently been used to isolate CTCs^[^ ^14^ ^]^; however, CTCCs locate closer to the RBC layers due to their large size and fast sedimentation speed, thus compromising yield when the buffy coat layer is targeted for extraction. Additionally, prolonged centrifugation time and high shear stress are likely to break apart CTCCs. An optimal CTCC apheresis technology should continuously isolate cell clusters (>2 cells) to a clean buffer with high yield from liters of undiluted blood, while minimizing shear stress and device resident time to preserve the integrity of CTCCs, and safely return the remaining blood components to the patient without dilution. Compared to the lossy bulk separation,^[^ ^15^, ^16^ ^]^ microfluidic devices offer superior precision to manipulate target cells at the microscale and have demonstrated high‐yield isolation of CTCs from blood.^[^ ^17^, ^18^ ^]^ Specifically, isolation strategies focus on differences between CTCs and white blood cells (WBCs), including the presence of epithelial cellular adhesion molecule (EpCAM) on CTCs (i.e., positive selection), the absence of WBC‐antigens on CTCs (i.e., negative selection), and physical attributes such as size.^[^ ^19^, ^20^ ^]^

Positive selection has been explored for CTC apheresis using intravascular indwelling wires and microchannels.^[^ ^21^, ^22^ ^]^ However, the yield and throughput are constrained by a need for CTCs to contact the limited anti‐EpCAM capture surface. Additionally, EpCAM expression of CTCs varies during cancer progression (i.e., epithelial‐to‐mesenchymal transition or EMT),^[^ ^23^, ^24^ ^]^ and is absent in non‐epithelial cancers. Negative selection^[^ ^14^, ^25^ ^]^ requires the addition of magnetic beads into the bloodstream to label WBCs, and will be lossy as CTCCs can include leukocytes.^[^ ^4^ ^]^ Size sorting^[^ ^19^, ^26^, ^27^, ^28^, ^29^, ^30^, ^31^ ^]^ is a promising approach for apheresis given the larger size of CTCCs. Mechanisms for size sorting include acoustic focusing,^[^ ^32^ ^]^ dielectrophoresis,^[^ ^33^ ^]^ deterministic lateral displacement (DLD),^[^ ^34^ ^]^ filtration,^[^ ^30^ ^]^ and inertial focusing.^[^ ^35^, ^36^, ^37^, ^38^, ^39^ ^]^ However, for continuous separation, besides substantial dilution of blood, the goals of high throughput and low shear stress oppose one another due to the small size of microfluidic channels (Supplementary Table S1). Microfluidic filtration using geometrical restrictions can process undiluted blood at low shear stress. Nevertheless, trapped CTCCs are exposed to extended device resident time and mechanical deformation under flow, compromising cluster integrity.^[^ ^40^, ^41^, ^42^, ^43^, ^44^ ^]^ To our knowledge, no existing technique has demonstrated continuous isolation of ultrarare CTCCs from undiluted blood with high yield, high throughput, and low shear stress (Table S1, Supporting Information).

Here, we report the Precision Apheresis for Non‐invasive Debulking of cell Aggregates (PANDA) microfluidic chip for continuous label‐free size sorting from whole blood. Critically, our device provides ≤15 Pa shear stress to retain intact clusters morphology, and uses a size cutoff of 15 µm,^[^ ^7^ ^]^ therefore ensuring isolation of the smallest CTCCs. The PANDA chip features two inlets (blood and buffer) and two outlets (returned blood and product containing CTCCs). Besides recovering >90% of spiked ≈24 HeLa cell clusters from 100 mL whole blood (≈500 billion blood cells) at 1 L h^−1^ throughput, the device residence time is under 20 seconds. Compared with the input blood, we observed similar plasma free hemoglobin concentration, WBC viability, and no increased activation of neutrophils or platelets (PLTs) in the returned blood. Notably, >99.9% of blood components including the PBMCs are returned, and only 12.5 mL of saline is added per 100 mL input blood, showing great promise for clinical apheresis to radically expand the access to ultrarare CTCCs.

## 2 Results

### 2.1 Design of PANDA Chip

**Figure 1:**
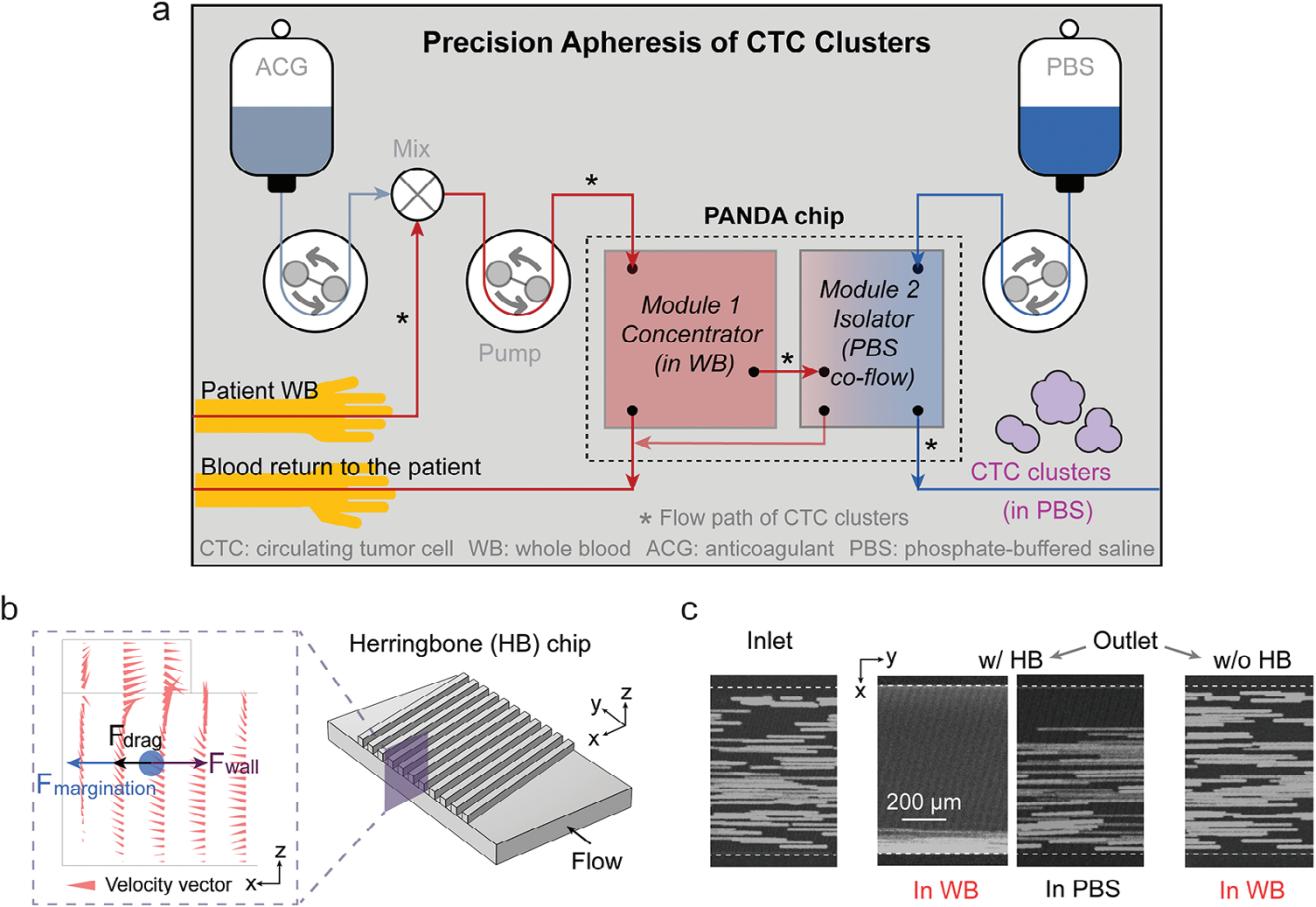
Precision apheresis of circulating tumor cell clusters (CTCCs). a) Whole blood from the patient is continuously drawn, mixed with anticoagulant, passed through the microfluidic device for CTCC isolation, and returned to the patient. b) With slanted grooves, herringbone chip focuses the target cells within whole blood toward one side of the channel. The inset shows that, along the x direction, the target cell experiences drag force induced by secondary flow, margination force exerted by red blood cells, and lift force from the wall. c) Streak images of fluorescent HeLa cells at the inlet (left) and comparison of cell focusing at the outlet of three cases: whole blood (WB) in herringbone channel, PBS in herringbone channel, and whole blood in rectangular channel. The channel height is 70 µm. Representative images of similar results are shown.

Our vision entails a precision apheresis process for CTCCs wherein blood is drawn from the patient, mixed with an anticoagulant, and subsequently passed through a microfluidic device to isolate CTCCs into PBS, followed by the return of sorted blood to the patient (**Figure** **1**a). Specifically, the microfluidic device needs to: 1) isolate CTCCs into PBS in a continuous, high throughput, and low shear stress manner by removing blood components without losing or breaking clusters; and 2) safely return the blood to patients without inducing alterations in blood, such as activating sensitive blood cells or causing excessive blood dilution. To mitigate the risk of clogging, the minimal channel depth is set to be 100 µm. The peak shear stress is maintained at 15 Pa to prevent breaking apart clusters.^[^ ^7^ ^]^

Label‐free size based sorting was adopted given the majority of blood cells including RBCs, WBCs, and PLTs are smaller than 15 µm. The microfluidic device consists of two modules: the concentrator (Module 1 in Figure 1a) that concentrates CTCCs in whole blood into a reduced volume; and the isolator (Module 2) that dilutes the product of Module 1 with buffer to reduce viscosity, which then allows size‐based separation of CTCCs into clean buffer using our previously developed nonequilibrium inertial separation array (NISA) technology.^[^ ^7^, ^20^ ^]^ Critically, the concentrator needs to enrich CTCCs with high yield in undiluted blood despite the extreme crowding of RBCs (i.e., ≈5 billion mL^−1^).

We addressed this challenge by leveraging the margination phenomenon, wherein RBCs preferentially migrate to the center of the blood vessels, thereby displacing WBCs and PLTs toward the wall.^[^ ^45^, ^46^, ^47^ ^]^ When combined with a herringbone design that induces a unidirectional secondary transverse flow, larger sized cells can be effectively focused to one side of the channel. As depicted in Figure 1b, along the x direction, the drag force arising from the secondary transverse flow and the margination force exerted by RBCs collectively push the target cells toward the wall, with the equilibrium location achieved through the counteracting wall lift force. In a rectangular channel (i.e., 70 µm height) with the top herringbone layer (Figure 1c, labeled as “w/ HB”), we observed excellent focusing of HeLa cells (average size 17.3 ± 3.3 µm) spiked in whole blood. In contrast, the degree of focusing is diminished when HeLa cells are spiked in PBS, suggesting that the drag force alone is not sufficient to achieve focusing. Furthermore, when switching to a rectangular channel with the same dimensions but without the top herringbone layer (Figure 1c, labeled as “w/o HB”), we did not observe any focusing within whole blood, underscoring the synergistic effect of margination and secondary flow.

### 2.2 Optimization of Single Stage Concentrator

First, we characterized and optimized the single stage concentrator using whole blood spiked with HeLa cells. Channel width was set to 750 µm. Upon varying the width of the herringbone groove from 20 to 40 and 60 µm, we observed that HeLa cells entered the groove and migrated to the other side of the channel, leading to cell loss (Figure S1, Supporting Information). Indeed, narrower width of the groove (i.e., 20 µm) effectively prevented entry of HeLa cells, therefore enhancing the degree of focusing (Figure 1b). Additionally, when we increased the gap between adjacent grooves from 20 to 40 µm (i.e., less densely packed grooves), we noted a reduction in the degree of focusing. Similarly, decreasing the groove tilt angle from 75° to 55° and 35° resulted in a lower degree of cell focusing (Figure S1, Supporting Information). Smaller (i.e., <20 µm) groove widths and gaps were not explored in this study due to challenges in microfabricating large area of tightly packed features with high precision using SU‐8. Therefore, we used 20 µm groove width, 20 µm groove gap, and 75° tilt angle for further optimization.

**Figure 2:**
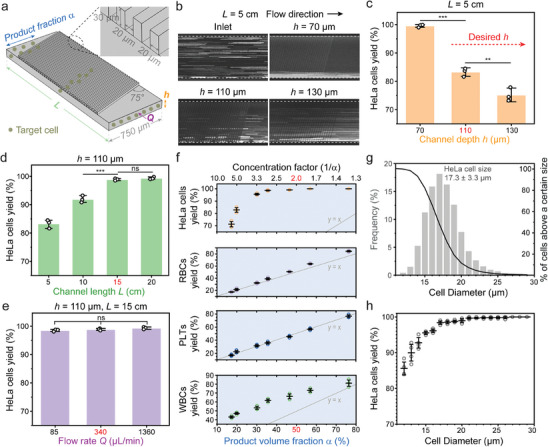
Optimization of single stage concentrator in whole blood. a) The key channel parameters for optimization. b) Comparison of streak images at the outlet for different channel depths. The inlet shows uniform distribution of HeLa cells across the channel width. The relatively low fluorescent intensity is related to light attenuation in whole blood and faster averaged flow velocity for deeper channels. Representative images of similar results are shown. c) The yield of HeLa cells for different channel depths. The other test conditions include that channel length is 5 cm, product fraction is 50%, flow rates are 137, 340, and 475 µL min^−1^ for 70, 110, and 130 µm channel depth respectively. The flow rate is adjusted to match the same peak shear stress of 15 Pa. d) The yield of HeLa cells for different channel lengths. The other test conditions include that channel depth is 110 µm, product fraction is 50%, flow rate is 340 µL min^−1^. e) The yield of HeLa cells for different flow rates. The other test conditions include that channel depth is 110 µm, channel length is 15 cm, product fraction is 50%. f) The yield of HeLa cells, red blood cells (RBCs), white blood cells (WBCs), and platelets (PLTs) for different product fractions. The other test conditions include that channel depth is 110 µm, channel length is 15 cm, flow rate is 340 µL min^−1^. g) Size distribution of HeLa cells and the percentage of cells that are above a certain size cutoff. h) The yield of HeLa cells with different sizes. The test conditions are that channel depth is 110 µm, channel length is 15 cm, flow rate is 340 µL min^−1^, and product fraction is 50%. Data are presented as mean ± s.d. for (c‐h), n = 3 for (c‐f), n = 4 for (h). One‐way ANOVA and Tukey's post hoc were used for statistical analysis. ns, *p* > 0.05; *p < 0.05; **p < 0.01; ***p < 0.001.

We then examined the effects of channel depth, channel length, flow rate, and product fraction on the yield of HeLa cells. With 5 cm channel length, the streak images indicated a decreasing level of focusing with increasing channel depth (**Figure** **2**b). In deeper channels, light attenuation increases in whole blood and averaged flow rate is faster (i.e., to maintain 15 Pa shear stress), therefore diminishing the intensity of fluorescent streak images. We quantitatively evaluated the sorting performance by direct measurements of HeLa cell yield from outlets. By taking a product volume fraction of 50% (i.e., 2x concentrator), we observed a decline in HeLa cell yield from 99.5% to 83.3% and 75.2% for channel depth of 70, 110, and 130 µm, respectively (Figure 2c). As a channel depth of >100 µm is desired to minimize clogging, we extended the channel length from 5 to 10, 15, and 20 cm using a channel depth of 110 µm, leading to an increased HeLa cell yield from 83.2% to 91.9%, 99.1%, 99.3% respectively (Figure 2d). To minimize pressure requirement, we chose 15 cm channel length. Additionally, when varying the blood flow rate from 85 to 340 and 1360 µL min^−1^, we found a consistent HeLa cell yield, suggesting that inertial force is not a dominant factor for cell focusing (Figure 2e). Thus, to maintain a peak shear stress of 15 Pa, 340 µL min^−1^ was used to screen different product volume fraction. By adjusting the outlet tubing resistance, we swept product volume fractions ranging from 17% to 77%, corresponding to 1.3x to 5.7x concentration factor (i.e., inverse of product volume fraction). As a result, HeLa cell yield remained high (>95%) for concentration factors below 3x but dropped rapidly to 71% as the concentration factor increased to 5.7x. Furthermore, due to the size‐based focusing nature of the process, larger WBCs are concentrated in the product. For instance, as the product volume fraction increased from 17% to 46% and 76%, the WBC yield increased from 43% to 66.3% and 81.1%, respectively. In the product, RBCs were slightly concentrated, while the concentration of platelets remained unchanged (Figure 2f). To ensure a high yield of HeLa cells and flexibility for parallelization, we selected the 2x concentration factor.

HeLa cell size distribution was measured using imaging flow cytometry after lysing RBCs (Figure 2g). We showed cell size dependent yield in Figure 2h. The single stage concentrator provided 95.5% yield of 15 µm cells and >99% for cells larger than 19 µm. We also tested two additional cell lines including MDA‐MB‐231 (15.5 ± 2.8 µm) and MGH‐BRx‐142 (20.4 ± 3.8 µm, Figure S2, Supporting Information). Using the cell size dependent yield (Figure 2h) and size distribution of spiked cells, we estimated the yields to be 99% and 95.3% for MGH‐BRx‐142 and MDA‐MB‐231 cells, which aligns with the experimentally measured yields of 99.7% and 93.3% respectively (Figure S2, Supporting Information). In addition, we validated that the HeLa cell yield remained high (i.e., ≈99%) across a wide range of hematocrit (HCT) levels in both ACD‐A (31.6 ≈ 42.8% HCT) and EDTA (33.9 – 48.7% HCT) blood (Figure S3, Supporting Information). Note that ACD‐A blood exhibits lower HCT levels due to dilution by anticoagulant (i.e., 1.5 mL ACD‐A to 8.5 mL blood).

### 2.3 Integration of Cluster Chip with Three‐Stage Concentrator

**Figure 3:**
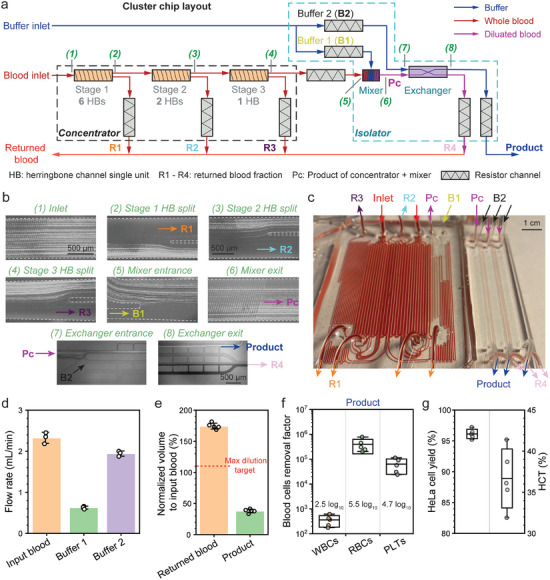
Integration of 3‐stage concentrator with the isolator. a) Layout of the cluster chip. The 3‐stage concentrator first concentrate target cells in whole blood. The concentrated product is diluted by buffer 1 using herringbone mixing, then injected into the exchanger along with buffer 2 to isolate target cells into clean buffer in Product outlet. All the other outlets are returned blood fractions, where R1, R2, R3 are whole blood and R4 is diluted blood. b) Streak images at different locations of the cluster chip. Representative images of similar results are shown. c) Picture of the cluster chip with labelled inlets and outlets. d) Measured flow rates for input blood, buffer 1, and buffer 2. e) Normalized volume (% of input blood) of the returned blood (i.e., R1 + R2 + R3 + R4) and product. f) The blood cells removal factor in product compared with input blood. g) Yield of HeLa cells in the product and hematocrit (HCT) of input blood. Data are presented as mean ± s.d. for (d‐e). Box and horizontal line represent standard deviation and mean respectively, whiskers represent max and min for (f‐g). n = 3 for (d), n = 5 for (e‐g).

Having optimized the single stage concentration, we designed the cluster chip by integrating a 3‐stage concentrator with the isolator to sort HeLa cells from whole blood to PBS (**Figure** **3**a). The process involves serial concentration of HeLa cells through six parallel stage 1 channels, followed by two parallel stage 2 channels, and one stage 3 channel before entering the isolator. In the isolator, we have two components including mixer and exchanger. Specifically, the blood was diluted with buffer fivefold in the mixer to reduce viscosity, ensuring a consistent size cutoff in the exchanger across a broad range of input blood viscosities. The same herringbone structure was used for mixing (i.e., Buffer 1 in Figure 3a) under low shear stress. For the exchanger, NISA technology employs inertial wall lift force to push cells away from walls of rectangular islands (450 µm long × 200 µm wide × 156 µm tall) in a size‐dependent manner, subsequently siphoning small‐sized cells (i.e., blood cells) through a 100 µm gap between one island and the next to achieve separation. The returned blood fractions from 3‐stage concentrator (R1, R2, R3) are undiluted blood while that from isolator (R4) is diluted blood. The flow was driven by a pressure source (i.e., 38 psi) and the resistor channels were designed accordingly to control the flow rate in each module.

Pressure‐induced inflation of microfeatures in soft PDMS devices will compromise the focusing performance. Therefore, we fabricated our device in rigid epoxy using an SU‐8 master mold patterned by photolithography (Figure 3b, details in the experimental section). Although the gap between two adjacent parallel channels is only 150 µm, we did not observe any leaking under pressure up to 80 psi. We monitored the focusing of HeLa cells at various critical locations of the cluster chip under the microscope. As depicted in Figure 3c, HeLa cells distributed uniformly across the channel width at the inlet, followed by successful focusing and concentration throughout the 3‐stage concentrator. The product from the concentrator is then diluted with Buffer 1, before entering the exchanger with Buffer 2. At the exit of the exchanger, we observed two distinct streams: one comprising HeLa cells in PBS and the other containing diluted blood. We showed 96.1% recovery of spiked HeLa cells in the product with input blood HCT levels ranging from 35.3% to 43.1%, demonstrating robust performance and successful integration of the concentrator and isolator.

In addition, we demonstrated 2.5 log_10_, 5.5 log_10_, and 4.7 log_10_ removal of WBCs, RBCs, and PLTs in the product from the input blood (Figure 3e). We also measured the flow rates of input blood, Buffer 1, and Buffer 2 to be 2.3, 0.6, and 1.9 mL min^−1^, respectively (Figure 3f). Using the pressure supply, flow rate varies depending on the input blood viscosity, while peak shear stress remains similar. Using 20 mL input blood, we found the volume of product and returned blood were 38% and 174% of the input blood, respectively. The returned blood volume exceeded our target (i.e., 110%) due to buffer dilution in the isolator, necessitating more stages of concentrator. Nevertheless, the cluster chip can be adapted to isolate CTCCs in unprocessed 20 mL peripheral blood samples from cancer patients within 10 min.

### 2.4 Reducing Dilution of Returned Blood with Six‐Stage Concentrator

**Figure 4:**
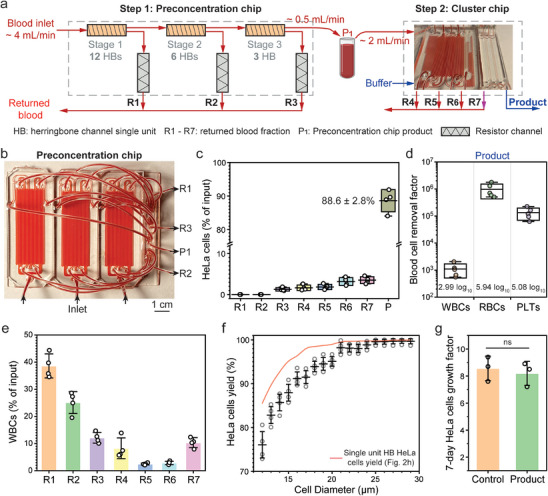
Performance of 6‐stage concentrator with the isolator. a) A two‐step process is used to reduce the returned blood dilution factor by adding a preconcentration chip before the cluster chip. The preconcentration chip is a 3‐stage concentrator, and the flow rates for input blood and product (P_1_) are approximately 4 mL min^−1^ and 0.5 mL min^−1^ respectively. P_1_ is collected and then fed into the cluster chip at approximately 2 mL min^−1^. b) Picture of the preconcentration chip with labeled inlets and outlets. c) Normalized percentage of HeLa cells (i.e., to the input) for different outlets. d) The blood cells removal factor in the product compared with input blood. e) Normalized percentage of white blood cells (WBCs) (i.e., to the input) for different outlets. The produce (P) is not shown due to extremely low value (<0.1%). f) The yield of HeLa cells with different sizes after 6‐stage herringbone concentrator and isolator. The red line represents the result for single stage concentrator. g) The 7‐day growth factor of isolated HeLa cells in the product, in comparison to untreated control cells. Box and horizontal line represent standard deviation and mean respectively, whiskers represent max and min for (c‐d). Data are presented as mean ± s.d. for (e‐g). n = 3 for (g), n = 4 for (c, e, f), n = 5 for (d). A two‐tailed paired t‐test was used to compare groups. ns, not significant, p > 0.05.

We next increased the stages of concentration from three to six to further reduce the dilution of returned blood and increase throughput. A 3‐stage preconcentration chip was designed with 12, 6, and 3 parallel concentrator channels in stages 1, 2, and 3, respectively (**Figure** **4**a). As shown in Figure 4b, the preconcentration chip includes three identical modules for convenient fabrication and scale‐up. The outlets were combined accordingly using an on‐chip connector. Under 15 Pa shear stress, flow rates for the input blood and product are 4 and 0.5 mL min^−1^, respectively. However, our cluster chip requires an input blood flow rate of 2 mL min^−1^. To validate the feasibility, we first ran 100 mL blood through the preconcentration chip and collected the product (P_1_ in Figure 4a) which was fed into the cluster chip in a separate run, with a total running time of 30 min.

We examined HeLa cell yield in each outlet from the preconcentration chip (R1 to R3) and cluster chip (R4 to R7 and product). We recovered 88.6% of spiked cells in the product (Figure 3c). Although R1 and R2 had negligible spiked cells, we noticed that cell loss increased as the sorting progressed through subsequent stages. Specifically, cell loss for R3 to R7 was 1.2%, 1.6%, 1.8%, 3.1%, and 3.5%, respectively. As illustrated in Figure 2f, large sized WBCs were also concentrated. Indeed, we observed increased WBCs concentration in outlets at later stages (Figure S4, Supporting Information). We speculate that overcrowding of WBCs can cause de‐focusing and loss of HeLa cells.

Compared to the cluster chip alone, we demonstrated a higher blood cell removal factor (3 log_10_, 5.9 log_10_, and 5 log_10_ for WBCs, RBCs, and PLTs respectively) in the product, indicating >99.9% return of blood component by combining R1 to R7 (Figure 4d). We further analyzed the relative percentage of WBCs across each outlet (Figure 4e). The 6‐stage concentrator returned 89.7% of total WBCs in undiluted blood (R1 to R6), and the exchanger (R7) returned 10.2% of total WBCs in diluted blood. The combined volume from R1 to R6 accounts for 98.1% of input blood. Therefore, to completely avoid dilution of returned blood, we can return only R1 to R6, excluding R7 (i.e., removing just 19 mL blood per liter processed). In addition, we found the overall cell size dependent yield dropped compared to the single unit concentrator (Figure 4f). Nevertheless, after the 6‐stage concentrator and the isolator, yield for 15 µm cells remains high at 88%. HeLa cells attached with different number of small‐sized WBCs were also observed (Figure S5, Supporting Information). We also cultured HeLa cells after sorting (details in Method sections). Isolated HeLa cells grew by 8.2‐fold after the 7‐day culture, which is not significantly different from untreated control cells (Figure 4g). In addition, we conducted in vitro scratch assay to assess cell migration capacity. Briefly, wound areas (i.e., without cells) were created by scratching the cell monolayer, then the surrounding cells migrated to fill up the wound areas over time (details in the Experimental Section). We observed similar migration capacity to close the wound area within 24 h using HeLa cells before versus after sorting (Figure S6, Supporting Information). Indeed, HeLa cells spend only 20 s in the microfluidic device with low shear stress.

### 2.5 Assessment of Returned Blood Properties

**Figure 5:**
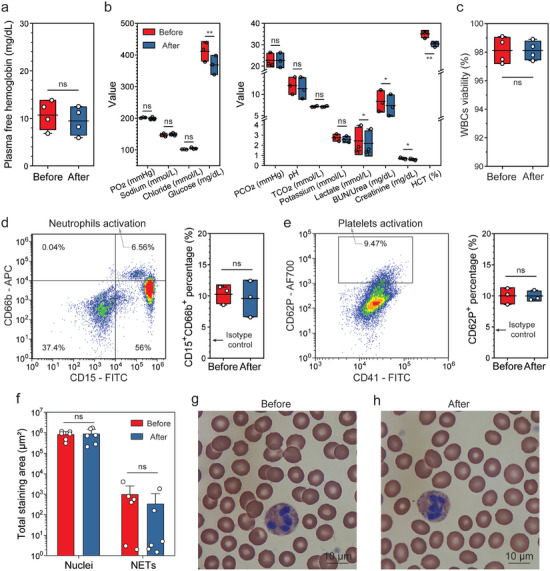
In vitro blood properties assessment before versus after microfluidic sorting. a) Comparison of plasma free hemoglobin levels. b) Blood chemistry measurement using i‐STAT CHEM8+ and CG4+ cartridges. c) Viability of white blood cells (WBCs) before and after sorting. d) Assessment of neutrophil activation using flow cytometry. Activated neutrophils are identified as CD15^+^CD66b^+^. e) Assessment of platelets activation using flow cytometry. Activated platelets are identified as CD41^+^CD62P^+^. f) Total staining area of neutrophil extracellular traps (NETs) and nuclei of WBCs using a microfluidic trap. Details can be found in the Methods section. g,h) Wright‐Giemsa staining of blood smears before and after microfluidic sorting. Representative images of similar results are shown. Box and horizontal line represent standard deviation and mean respectively, whiskers represent max and min for (a‐e). Data are presented as mean ± s.d. for (f). n = 3 for (b, d, e), n = 4 for (a, c), n = 6 for (f). A two‐tailed paired t‐test was used to compare groups. ns, not significant, p > 0.05, *p < 0.05; **p < 0.01; ***p < 0.001.

To assess whether microfluidic sorting altered in vitro blood properties, we ran a battery of tests on blood before (i.e., unprocessed) and after sorting (i.e., combining R1 to R7). Plasma free hemoglobin concentration showed no significant change, indicating minimal hemolysis caused by the sorting process. Blood chemistry was evaluated using cartridge‐based tests (CHEM8+ and CG4+, iStat, Abbott). Due to the PBS buffer dilution in R7, we observed the concentrations of glucose, lactate, urea, creatinine, and hematocrit decreased by ≈10% after sorting when normalized to pre‐sorting values (**Figure** **5**b; Figure S7, Supporting Information). This dilution can be avoided by not returning R7 as mentioned earlier. The gas pressure (CO_2_ and O_2_), pH, and concentrations of sodium, chloride, and potassium ions remained unchanged.

At the cellular level, we observed similar viability of WBCs after sorting (Figure 5c). We also tested whether microfluidic sorting induced activation of sensitive cells including neutrophils and platelets. Using flow cytometry, we showed that the percentages of activated neutrophils (i.e., CD15^+^CD66b^+^) and platelets (i.e., CD41^+^CD62P^+^) remained unchanged post sorting (Figure 5d,e; Figures S8 and S9, Supporting Information). In addition, we used a customized microfluidic device with micropillars to capture neutrophil extracellular traps (NETs) in whole blood (details in Experimental Section). We found that no additional NETs were generated due to the sorting (Figure 5f). To further examine the morphology of blood cells, Wright‐Giemsa staining of blood smears was performed. As presented in Figure 5g,h, red blood cells showed normal size, biconcave disc shapes, and pinkish‐red color with central pallor. Quantitatively, the diameter and circularity of RBCs are 7.97 ± 0.51 µm and 0.87 ± 0.01 before microfluidic sorting, and are 7.88 ± 0.46 µm and 0.86 ± 0.05 after microfluidic sorting, suggesting no statistical differences (Figure S10, Supporting Information). Neutrophils showed intact structures, and no NETs were observed. Similar morphologies of mononucleated cells were observed before versus after microfluidic sorting (Figure S11, Supporting Information). Indeed, blood cells experience low shear stress (<15 Pa) and short chip residence time (<20 s) during sorting, resulting in minimal change in blood properties.

### 2.6 Continuous Recovery of Spiked Rare Cell Clusters in Whole Blood

**Figure 6:**
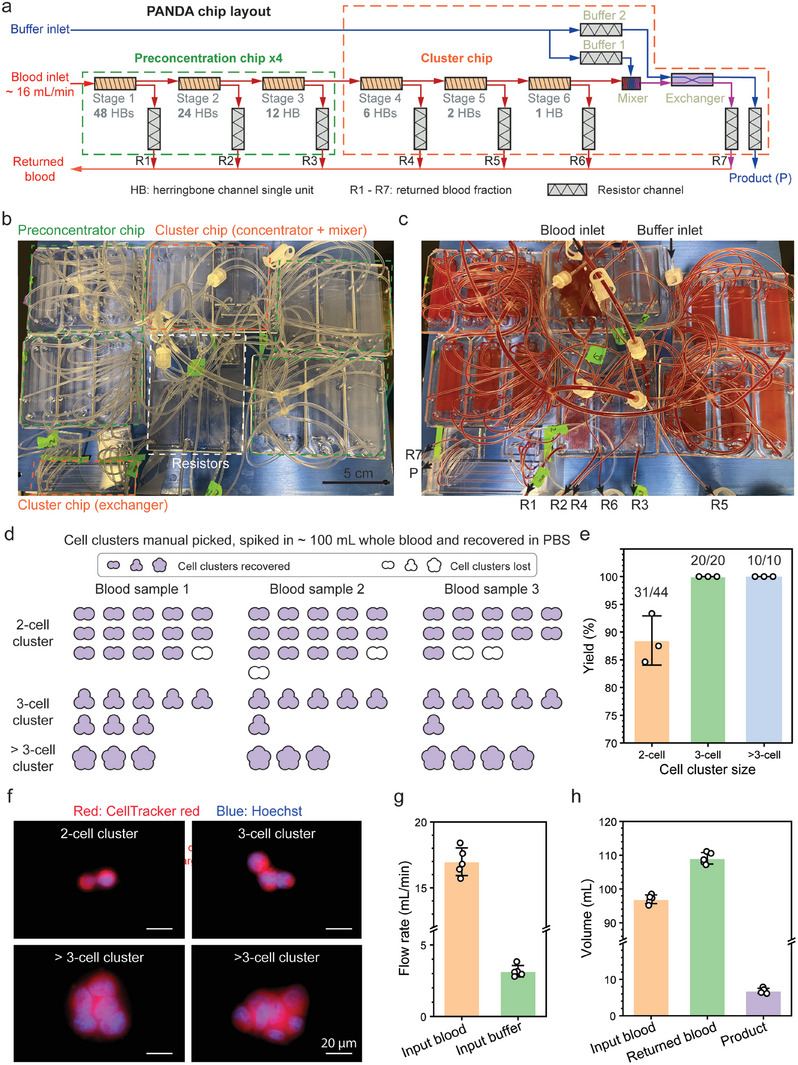
Continuous recovery of spiked rare cell clusters from whole blood using the PANDA chip at ≈1L h^−1^. a) The PANDA chip consists of 4 preconcentration chips and 1 cluster chip. b) Picture of PANDA chip before sorting. To reduce the device footprint, peak shear stress in resistor channel (white dash line) is 50 Pa, and 15 Pa elsewhere. c) Picture of PANDA chip during sorting, with labelled inlets and outlets. d) Recovery of rare HeLa cell clusters spiked in ≈100 mL whole blood in about 6 min. e) Yield of cell clusters. f) Images of recovered cell clusters with different sizes. g) Flow rate of input blood and buffer. h) Volume of input blood, returned blood (combining R1 to R7), and product. Data are presented as mean ± s.d. for (e, g, h). n = 3 for (e), n = 5 for (g, h).

Finally, we sought to demonstrate continuous recovery of spiked rare cell clusters in large volume (i.e., 100 mL) of undiluted blood at high throughput consistent with what would be used in a clinical apheresis. As shown in **Figure** **6**a, the PANDA chip integrates four preconcentration chips with one cluster chip. To reduce the device footprint for this experiment, maximum shear stress was increased to 50 Pa only in the resistor channels R1 to R6, while maintained at 15 Pa elsewhere (Figure 6b‐c). This continuous sorting process provides higher throughput and eliminates the risk of CTCC loss during transfer in the previous two‐step sorting approach (Figure 4a).

We manually picked and spiked ≈24 HeLa cell clusters into ≈100 mL blood and evaluated the recovery of clusters (Figure 6d). Cell clusters were categorized into 2‐cell, 3‐cell and >3‐cell clusters, with an overall yield of 31 out of 44 (88.5 ± 3.6%, n = 3), 20 out of 20 (100%, n = 3), and 10 out of 10 (100%, n = 3), respectively (Figure 6d,e). The recovered clusters showed different sizes and morphologies, demonstrating the robustness of our sorting device (Figure 6f). For the PANDA chip, flow rates of input blood and buffer were 17 ± 0.9 mL min^−1^ (measured by the running time for 100 mL blood) and 3.2 ± 0.3 mL min^−1^ (measured by a flow meter), respectively. With a throughput of ≈1 L h^−1^, 100 mL unprocessed blood can be processed within 6 min. In addition, the volume of returned blood (combining R1 to R7) increased by 12.5 ± 2% when normalized to the input blood volume (97 ± 1.1 mL), and the volume of product was reduced to 6.8 ± 0.6 mL. The total blood volume inside the microchannels is 1.4 mL.

## 3 Discussion

We have described a precision apheresis microfluidic device (PANDA chip) for continuous label‐free size sorting of CTCCs from undiluted whole blood, with low shear stress (≤15 Pa) and high yield (>90%) at 1 L h^−1^ throughput. Compared to single CTCs, CTCCs have distinct epigenetic markers, and are less apoptotic due in part to intact cell‐cell junctions that prevent anoikis, thereby playing a major role in metastasis. The number of CTCCs varies across different patient populations and can be less than two CTCCs in 20 mL of standard blood tubes. A generalized approach is increasing processed blood volumes (i.e., apheresis) to demystify the ultrarare yet clinically significant population of tumor cells. Although leukapheresis can isolate nucleated cells from entire blood volume of ≈5 L, harsh and lossy bulk centrifugation jeopardizes the morphology, integrity, and yield of CTCCs. No existing apheresis system specifically isolates intact CTCCs from liters of blood to the best of our knowledge.

Common strategies to isolate CTCCs ex vivo are based on physical attributes using a microfluidic device. For filtering/trapping mechanisms, Fatih et al. exploited shape difference to trap CTCCs with a microfluidic see‐saw geometry.^[^ ^48^ ^]^ However, blood throughput was only 2.5 mL h^−1^. Microfluidic filters like Parsortix can process blood at low shear.^[^ ^40^ ^]^ Yet tumor cells must then endure entrapment under confined flow in filter pores for hours of processing. This leads to loss since cell clusters can squeeze through surprisingly small pores if given enough time.^[^ ^41^ ^]^ In addition, cells can be damaged by passing through the 6–7 µm pores, and mutations can occur if the nucleus is mechanically squeezed in transit,^[^ ^42^, ^43^ ^]^ problematic for trapped tumor cells with high nucleus to cytoplasm ratio. These limitations also persist in the grid‐based filter which can have high blood throughput at low shear stress, such as in Boya et al.^[^ ^44^ ^]^ For continuous size‐based separation mechanisms, Au et al used DLDs to sort CTCCs at low‐shear,^[^ ^49^ ^]^ but DLDs are prone to clogging at higher flow rates due to bumping.^[^ ^38^, ^50^ ^]^ The vortex chip is fast,^[^ ^51^ ^]^ but peak wall shear stress is extremely high (up to ≈1000 Pa), risking cluster integrity. Inertial focusing using spirals can operate at low shear stress,^[^ ^36^, ^52^ ^]^ yet spirals require RBC lysis or large dilution of blood to retain yield.^[^ ^15^ ^]^ Enabled by the NISA technology, Edd et al. reported isolation of spiked rare CTCCs with high yield (>80%) from blood at 30 mL h^−1^ and ≤15 Pa shear stress, albeit requiring 1:1 dilution of input blood.^[^ ^7^ ^]^ Altogether, all of these platforms for CTCC sorting encounter significant hurdles when applied to apheresis, which necessitates high throughput, high yield, low shear stress, minimal dilution of returned blood, and continuous isolation simultaneously. Our work bridges this critical gap by enabling label‐free microfluidic CTCCs apheresis to overcome the challenge of insufficient and/or variable recovery of CTCCs across broad ranges of patient populations using small volumes of blood (i.e., up to 20 mL).

In our work, we highlighted several significant advances. First, to minimize blood dilution, we achieved continuous sorting of CTCCs from undiluted blood (i.e., no trapping of CTCCs followed by extended collisions with blood cells in filtration‐based approaches), which has not been demonstrated in the existing literature. We revealed size‐dependent focusing attributed to synergies between secondary transverse flow and margination. Such cell focusing ability in a tall (110 µm) and wide (750 µm) channel enables high blood flow rate at low shear stress without clogging. In addition, we showed that HeLa cell yield remained high (92.1%) even after 6 stages of consecutive concentration (>60‐fold) in whole blood. Future investigations into the detailed focusing mechanisms are needed. Second, for continuous CTCC isolation at 1 L of blood per hour, we created an integrated PANDA chip consisting of 6‐stage concentration in blood, inline mixing with buffer to reduce viscosity, and size‐based NISA separation into clean buffer. To avoid deformation of microchannels under pressure, we fabricated epoxy‐based rigid devices. Notably, even with a bonding area as narrow as 150 µm in width, no leakage was observed under pressure up to 80 psi. Plastic chips from cyclic olefin copolymer (COC) can be readily mass produced and avoid most of the connection tubing (Figure 6b) for seamless integration in the future. The device resident time for blood cells is <20 s and dead volume of the microchannels is only 1.4 mL, which eases return of unaltered blood and enables apheresis for young patients where extracorporeal blood volume limitations are stringent. Third, spiked rare cell clusters were recovered with high yield and without breakage using the PANDA chip, averaging a sorting speed of 1.4 billion cells per second. Those outcomes demonstrated the robustness of our technology. In contrast, bulk spiking of cell clusters was often used to evaluate cluster recovery in most other studies, prohibiting assessment regarding the yield of CTCCs in different morphologies and whether CTCCs break apart during sorting.^[^ ^44^, ^48^, ^49^ ^]^

We note that 10–20 mL patient blood is not suitable for the PANDA chip which is designed to process large blood volumes. Specifically, in the current form of epoxy device, total dead volume is 4.4 mL (i.e., 1.4 mL from the microchannels and 3 mL from tubing connections). In addition, after priming the chip with PBS, it takes ≈25 s for blood to replace PBS to allow stable CTCCs sorting performance. Meanwhile, blood is supplied at ≈16 mL min^−1^ and 6.6 mL is consumed. Hence, a total of 11 mL (i.e., 4.4 + 6.6) blood will not yield reliable CTCCs isolation. Future work involving large volumes of blood from cancerous patients and/or animal models is needed.

One limitation of our present study is the absence of in vivo validation with an animal model. Establishing the safe return of sorted blood is crucial for the potential clinical translation of our findings. The in vitro assessment of blood properties conducted in our work may not fully capture the dynamic performance when blood is directly sourced from the circulation. The design of our PANDA chip emphasizes high throughput, rendering a mouse or rat model impractical due to their comparatively small total blood volume. A large animal model such as dog or pig will provide an invaluable platform to rigorously evaluate the safety and efficacy of our technology under physiological conditions.

With the potential to yield over 100 times more live clusters compared to current methods, the label‐free precision apheresis for CTCC from liters of peripheral blood is likely to enable reliable isolation of the most metastatic cell population in cancer patients. This will provide exciting opportunities to understand the metastasis process, and further transform the diagnosis, management, and treatment of cancer patients. For instance, in targeted therapy, an urgent need is to test a patient's own tumor cells ex vivo, before and during treatment, to predict drug response/resistance and monitor efficacy of each treatment cycle. As CTCCs are known to be not apoptotic like single CTCs often are, gentle sorting of large numbers of CTCCs will facilitate personalized oncology by ex vivo culture for real time functional drug testing of cancer patients. Moreover, if alternatively applied to routine collection of up to a pint (i.e., 473 mL) of blood in high‐risk patient populations, our technology readily allows rapid isolation of live CTCCs and large‐sized CTCs in <30 min. Importantly, the sorted blood remains minimally altered and can be used for downstream single CTC isolation (i.e., negative sorting) and cell‐free DNA analysis. Together, this strategy offers a non‐invasive approach for early detection where regular 20 mL blood tubes could fail to provide any CTCCs. Furthermore, beyond CTCCs, due to the size sorting mechanism of our PANDA chip, our technology can be employed to isolate many other larger cells and clusters in circulation for a range of applications.

## 4 Experimental Section

### Blood Samples

Most healthy donors blood samples were purchased from Research Blood Components, LLC (Brighton, MA). Blood samples were also acquired from healthy internal donors following protocols approved by the Massachusetts General Hospital IRB (protocol numbers 2009‐P‐000295 and 2015‐P‐000656). All samples were obtained following the applicable guidelines.

### Device Fabrication

The two‐layer herringbone structures were first fabricated on a silicon wafer using negative photoresist (SU‐8, MicroChem) via standard photolithography. The channel and herringbone dimensions were measured with a surface profilometer (Veeco Instruments Inc). Then PDMS (Sylgard 184) was poured, degassed, and cured using SU‐8 as the master mold. For the single stage herringbone concentrator and the exchanger, the cured PDMS was cut, and holes were punched using a biopsy punch. Channels were enclosed by oxygen plasma treatment, followed by bonding to a glass slide (1 × 3 inch2) treated by oxygen plasma. For the cluster chip and preconcentration chip, the PDMS was cut into the desired size for the rigid epoxy device fabrication, as detailed elsewhere.^[^ ^53^ ^]^ Briefly, the PDMS replica was first treated with oxygen plasma and quickly placed in a vacuum chamber together with tricholorperfuorooctylsilane (TCPFOS, Sigma) for 30 min. Next, using the surface coated PDMS as the new master, PDMS was poured, degassed, and cured to make the PDMS_epoxy mold for epoxy casting. Holes were punched in the PDMS_epoxy mold. The Teflon rod‐tubing complex was inserted into punched holes for tubing connection to the epoxy device. The epoxy (EpoxAcast 690) was mixed, degassed, and poured into the PDMS_epoxy mold. After curing for 22 h at room temperature, the epoxy microfluidic device was released, bonded to an epoxy coated glass slide (4 × inch2) and left at room temperature for 24 h before use. To make the epoxy coated glass slide, a PDMS_glass mold was first created by pouring and curing PDMS around the glass slide. Next, the epoxy was mixed, degassed, and poured into the PDMS_glass mold, and sealed with a glass slide. After curing the epoxy at room temperature for 24 h, the epoxy coated glass slide can be used to bond the epoxy microfluidic device.

### Cell Culture and Preparation

HeLa cells and MDA‐MB‐231 (ATCC) were cultured in Dulbecco's Modified Eagle Medium (DMEM) supplemented with 10% fetal bovine serum (FBS) and 1% penicillin/streptomycin (P/S), at 37 °C and 5% CO_2_. Media was changed every 3–4 days and cells were passaged when reaching 80% confluent. As previous described,^[^ ^7^, ^49^ ^]^ BRX‐142 was initially isolated from a patient and cultured in ultralow attachment dishes in RPMI‐1640 media supplemented with 20 ng/mL epidermal growth factor, 20 ng mL^−1^ basic fibroblast growth factor, 1X B‐27 supplement, and 1X Antibiotic‐Antimycotic at 37 °C, 5% CO_2_ and 4% O_2_. For spiking experiments, cells were detached from culture flasks with 0.25% Trypsin/ 0.53 mM EDTA solution, then pelleted and resuspended in media. The resuspended cells were pipetted several times to break large cell clumps. Cells were stained with Hoechst (1:1000) and Celltracker Red (1:1000) for 30 min in the incubator (37 °C and 5% CO_2_). Stained cells were washed three times by centrifuging at 200 g for 5 min and resuspended in PBS. For HeLa cell yield experiments, stained cells were spiked in whole blood at a density of 5000 cells mL^−1^. For rare cell cluster spiking experiments, clusters of different sizes were manually picked and counted using a single cell picker, then transferred into large volume of blood using a pipette. For large cell clusters with 3D structures which are difficult to estimate total cell number, they were categorized into the group of >3 cell clusters.

### Microfluidic Device Operation

For the single stage herringbone concentrator made in PDMS, the device was first primed with 0.2% Pluronic F68 (ThermoFisher Scientific) in PBS to minimize non‐specific cell sticking. The whole blood with spiked HeLa cells was loaded into a 10 mL syringe (BD Bioscience). The flow was driven by a syringe pump (Harvard Apparatus). The product was collected and weighed to obtain the product fraction. To vary the product fraction, the produce/waste resistance was adjusted by adding 0.01 inch inner diameter PEEK tubing (Fisher Scientific) with different lengths. Streak images were taken with an upright fluorescent microscope (Nikon Eclipse 90i).

For the cluster chip, preconcentration chip and apheresis chip that were made in epoxy, a pressure source (Flow EZ, Fluigent) was used to drive the flow. The device was primed with 0.2% Pluronic F68 before use. Each outlet was collected and weighted separately. To measure the blood flow rate, the input blood volume and device running time were recorded. For the buffer flow rate, Sensirion flow meters (SLI‐1000 for <1 mL min^−1^ and SLF35‐1300F for >1 mL min^−1^) were connected into flow path of buffer 1 and buffer 2 (Figure 3a). The cluster chip, preconcentration chip, and apheresis chip were operated at 38 psi, 20 psi, and 80 psi respectively.

### HeLa Cell Yield and Microfluidic Device Performance

For the bulk HeLa cells spiking experiment, 10 µL was taken from each outlet and the number of HeLa cells was counted using disposable hemocytometer slides (Fisher Scientific) under a 4x objective lens. More than 10 views were included. HeLa cell yield was then calculated using the volume fractions of each outlet based on the weight measurement. For the rare HeLa cell cluster spiking experiment, post isolation by the apheresis chip, the product fraction (in PBS) was collected in a 6‐well plate, and cells were allowed to settle to the bottom for 45 min at room temperature. The pre‐stained cell clusters were identified by panning the microscope objective (20x) throughout the well using an inverted microscope (Nikon TiE). Only cells with both positive Hoechst and CellTracker Red staining were counted. The yield of cell clusters was calculated from the known number of spiked cell clusters.

The size dependent HeLa cell yield was measured using imaging flow cytometry (Amnis ImageStream MK II). Specifically, for outlets with blood cells (i.e., except the Product outlet which is in PBS), RBCs were lysed using lysis solution (Miltenyi Biotech) for 20 min at room temperature, followed by centrifuging at 200 g for 5 min to pellet the nucleated cells. The HeLa size distribution of each outlet was then measured by the imaging flow cytometry. Based on the volume fractions of each outlet, the size dependent yield can be calculated.

To obtain the fractions of blood cells (RBCs, WBCs, PLTs) in each outlet (Figures 2f and 4e), a complete blood count (CBC) was performed using a KX‐21N Hematology‐Analyzer (Sysmex). The blood cell fractions were then calculated using the volume fractions of each outlet based on the weight measurement.

The blood cell removal factors in the final product were determined by analyzing 10 µL final product in a disposable hemocytometer slide, after staining the WBCs with Hoechst (1:1000). Briefly, the number of RBCs, WBCs and PLTs were counted to obtain the concentrations. The blood cell removal factors can be calculated from the volume of the product and input blood, and RBCs, WBCs, PLTs concentrations of the input blood.

To evaluate the proliferation of HeLa cells after microfluidic sorting, overnight UV treatment was performed to sterilize the microfluidic device and associated components (i.e., tubing, Luer connectors, etc). We spiked 500 000 fluorescently labeled (i.e., CellTracker Red) HeLa cells into 100 mL blood and collected the product (i.e., ≈7 mL in PBS). We then centrifuge and resuspend the product in 1 mL HeLa cell culture media. We calculated the total number of recovered HeLa cells using a hemocytometer under the fluorescent microscope. Then, the recovered HeLa cells were plated into two T25 flasks for culture, with media change every other day. After 7 days, HeLa cells were harvested to obtain the total cell counts to calculate the proliferation. In the control group, same cell culture procedures were employed using fluorescently labelled HeLa cells without microfluidic sorting.

To further assess the migration capacity of HeLa cells post microfluidic sorting, we performed in vitro scratch assay. Specifically, HeLa cells in the product were resuspended in culture media and seeded into 24‐well plate at a density of 1 × 10^5^ cells per well for culture. After reaching ≈90% confluency, a 200 µL pipette tip was used to scratch the cell monolayer to create the wound intersection in the shape of a cross. Cell debris was carefully removed by rinsing with media several times. Next, low serum culture media (i.e., 5% FBS in DMEM) was added for culture to reduce cell proliferation. Images of the wound intersection were taken at different time points to measure its area change due to cell migration.

### Blood Property Assessment before versus after Microfluidic Sorting

The input blood (≈100 mL) was first run through the preconcentration chip. The product (in whole blood) was collected, then run through the cluster chip. This is to ensure that peak shear stress experienced by blood cells is less than 15 Pa, as part of the apheresis chip provides 50 Pa shear stress. All the outlets except the product were pooled and referred to as the returned blood (R1 to R7 in Figure 4a). The input blood was compared with the returned blood using the assays described below.

To measure the plasma free hemoglobin levels, 100 µL of the input and returned blood were each diluted tenfold with PBS, then centrifuged at 500 g for 5 min. Concentration of hemoglobin in the supernatant was determined by measuring absorbance at 380, 415, and 450 nm (BioTek HT Microplate Reader), and calculated as C_hemo_ = 1.68 A_415_ – 0.84A_380_‐0.84A_450_ according to the Allen correction formula,^[^ ^54^, ^55^ ^]^ where C_hemo_ is the hemoglobin concentration, A_415_, A_380_, A_450_ are the absorbance at 415, 380, and 450 nm respectively. PBS was used as the blank controls.

WBC viability was measured using live/dead fluorescent staining. Specifically, 10 µL of the input and returned blood were each mixed with Hoechst (1:1000) and propidium iodide (PI, 1:1000) and incubated at room temperature for 20 min in the dark. The blood was diluted fivefold with PBS and loaded into a disposable hemocytometer. A total of 20 random views were taken with a 10x objective.

Blood chemistry cartridges including i‐STAT CHEM8+ and CG4+ (Abbott) were used following the product instructions. After adding ≈90 µL blood, the cartridge was inserted into a point of care i‐STAT blood chemistry analyzer (Abbott). The measured values were compared for input blood and returned blood.

The amount of neutrophil extracellular traps (NETs) was measured from whole blood using a customized microfluidic device without centrifugation or RBC lysis, as detailed elsewhere.^[^ ^56^ ^]^ Briefly, the microfluidic device includes a closely packed array of posts to mechanically capture the NETs with low shear stress (<2 Pa) as blood flows through. The blood was mixed with Sytox green (1:1000, for staining NETs) and Hoechst (1:1000), and incubated for 20 min at room temperature in dark. Next, blood was diluted with PBS by 10‐fold and introduced into the microfluidic device. Images were taken with an inverted microscope (Nikon TiE) and areas of NETs and nuclei were quantified using ImageJ software.

Blood smears were prepared for the Wright‐Giemsa staining. Specifically, 5 µL blood was deposited on the microscope slide and smeared using a cover glass, followed by air dry. Next, the slide was rinsed with methanol for 30–60 seconds. After draining excess methanol from slide, 1 mL Wright‐Giemsa solution (Fisher Scientific) was added to cover the entire slide and allow cells to stain for 2 min. Then 1.5 mL PBS was added to the staining solution and mixed by gently rocking the slide for 1 min. The mixture was left on the slide for an additional 2 min. The slide was then rinsed with deionized water and allowed to air dry. Images were taken with oil immersion 100x objective. The diameter and circularity of RBCs were quantitatively analyzed using ImageJ software.

To assess platelet and neutrophil activation, imaging flow cytometry (Amnis ImageStream MK II) was used. For platelets, 100 µL blood was mixed with Alexa Fluor 700 CD62P antibody (1:20, clone AK4, BioLegend) and FITC CD41 antibody (1:20, clone HIP8, BioLegend), and incubated at room temperature for 30 min in the dark. The blood was then diluted 50‐fold using PBS and analyzed using the imaging flow cytometer with a 40x objective. Isotype control was performed using Alexa Fluor 700 mouse IgG (1:20, clone MOPC‐21, BioLegend). For neutrophils, 100 µL of blood was mixed with APC CD66b antibody (1:20, clone G10F5, BioLegend), FITC CD15 antibody (1:20, clone HI98, BioLegend) and Hoechst (1:1000), and incubated at room temperature for 30 min in the dark. Then the Fix/Lyse solution (Thermofisher) was added, mixed, and incubated for 20 min at room temperature in the dark to simultaneously lyse the RBCs and fix the remaining WBCs. The fixation is to avoid further activation of neutrophils in subsequent centrifugation and washing steps. Next, the cells were centrifuged, washed three times, and resuspended in PBS before analyzed by the imaging flow cytometry. Isotype control was performed using APC mouse IgM (1:20, cloneMM‐30, BioLegend).

### Computational Fluid Dynamics Model

The computational fluid dynamics model of the herringbone channel for Figure 1b was performed with COMSOL. The channel depth is 70 µm, width is 750 µm. The height, width, gap, and angle of the groove are 30 µm, 20 µm, 20 µm, and 75°, respectively. The blood viscosity was assumed to be 4 × 10^−3^ Pa×s for simplicity. Inlet flow rate is 70 µL min^−1^ to ensure 15 Pa peak shear stress. Outlet pressure was set to 0. The 3D model has ≈3 million tetrahedral mesh elements.

### Statistics

Experimental data was presented as mean ± s.d. unless otherwise specified. Sample size (n) was included in the figure captions. For Figures 1, 2, 3c, and 5g,h, and 6f, representative images were shown from n = 3 experiments with similar results. Paired two‐tailed t‐test and one‐way ANOVA with Tukey's post hoc were used for statistical analysis using Excel. The p‐values >0.05 were considered statistically non‐significant (ns). The p‐values <0.05 were considered statistically significant, with *p <0.05, **p <0.01, and ***p <0.001.

## Conflict of Interest

The authors declare no conflict of interest.

## Supporting information

Supporting Information

## Data Availability

The data that support the findings of this study are available from the corresponding author upon reasonable request.
