# Supplementary material for: Label‐Free Microfluidic Apheresis of Circulating Tumor Cell Clusters
Source: Adv Sci (Weinh). 2024 Aug 28;11(40):2405853. doi: 10.1002/advs.202405853 (PMC11515904; doi:10.1002/advs.202405853)
Supplement: Supplementary file 1 — Supporting Information [file ADVS-11-2405853-s001.docx]

**Supplementary Materials**

**Label-free microfluidic apheresis of circulating tumor cell clusters**

Li Zhan^1,2^, Jon Edd^1,3^, Avanish Mishra^1,2,3^, Mehmet Toner^1,2,4*^

1 Center for Engineering in Medicine and Surgery, Massachusetts General Hospital, Boston, MA, USA

2 Harvard Medical School, Boston, MA, USA

3 Cancer Center, Massachusetts General Hospital, MA, USA

4 Shriners Children, Boston, MA, USA

Corresponding author email: mtoner@mgh.harvard.edu

**Table S1**. **Comparison of cell cluster isolation methods in the context of apheresis^1-10^**


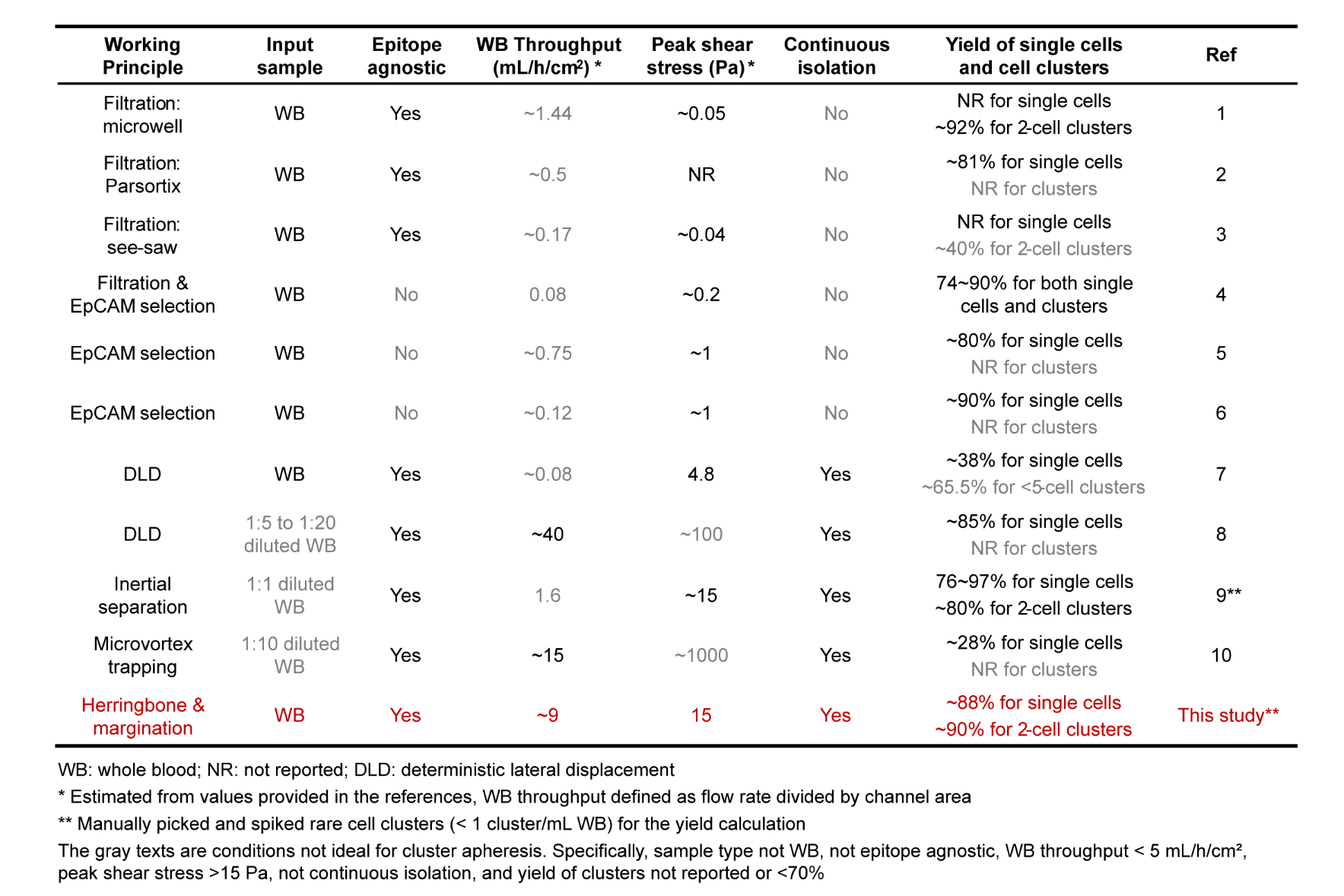


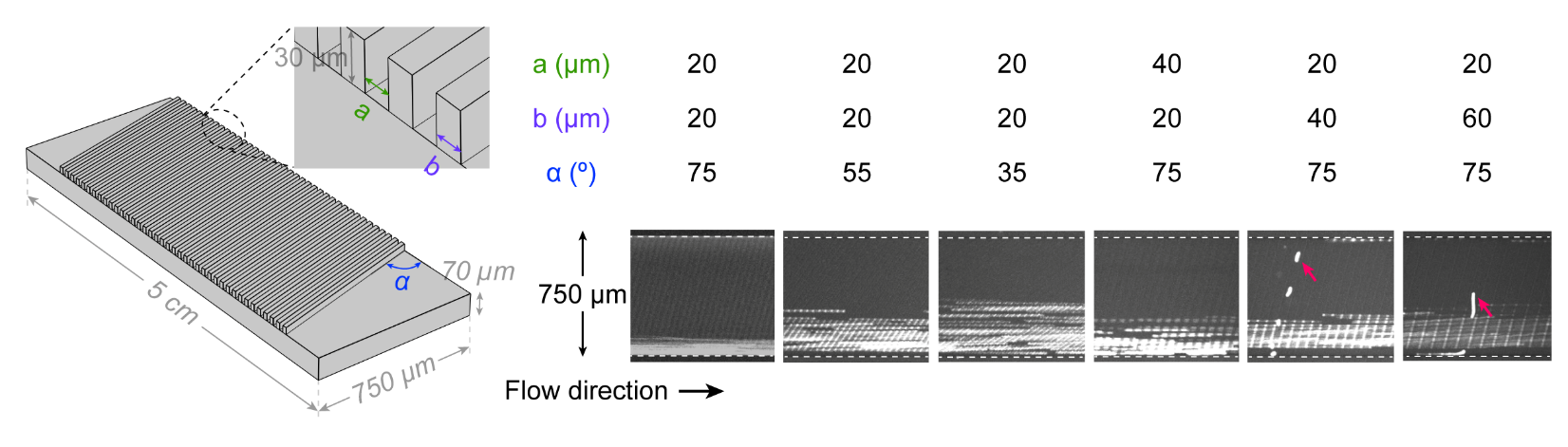


**Fig. S1.** Optimization of the groove dimensions for HeLa cell focusing within whole blood. The height of the groove is 30 µm. Streak images of HeLa cells inside the channel were shown. Red arrows indicate the entry of cells into the groove.


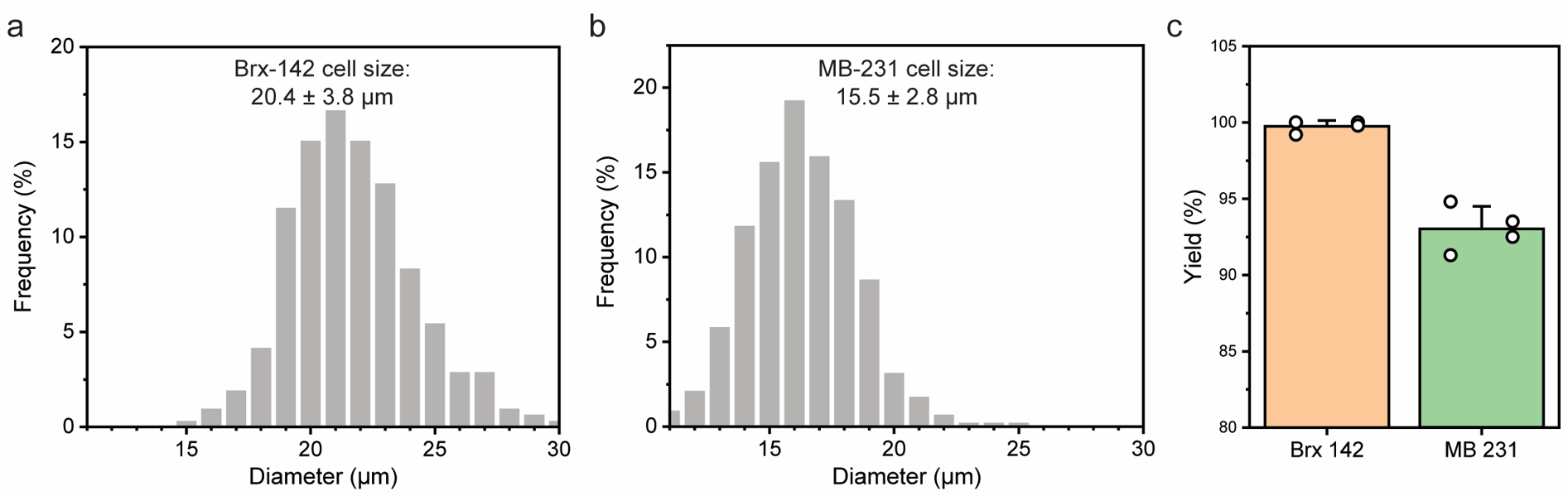


**Fig. S2.** Validation of the concentrator performance with different cell lines. **(a-b)** Cell size distribution measured by imaging flow cytometry. **(c)** Yield of Brx-142 and MB-231 cells in whole blood using single stage 2x concentrator. Channel height is 110 µm, channel length is 15 cm, and flow rate is 340 µL/min.


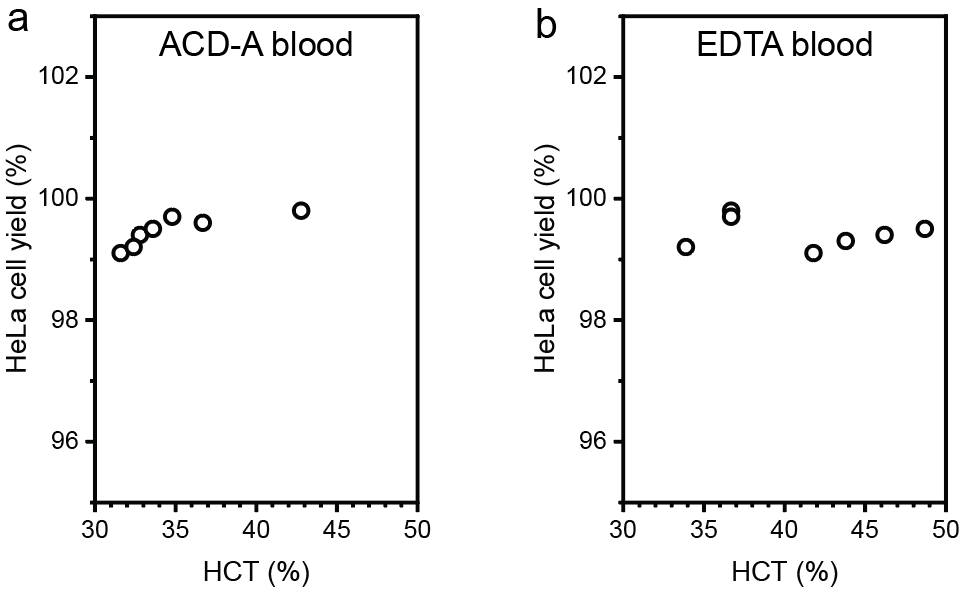


**Fig. S3.** Yield of HeLa cells using **(a)** ACD-A and **(b)** EDTA blood with different hematocrit (HCT) levels using single stage 2x concentrator.


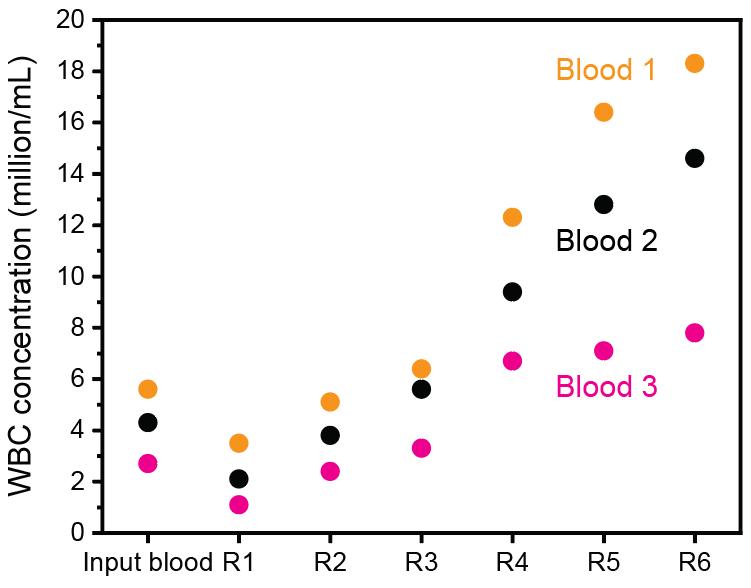


**Fig. S4.** The concentration of white blood cells (WBCs) in different stages of the returned blood fractions. In the 6-stage concentrator, R1 to R6 are undiluted blood, as illustrated in Fig. 4.


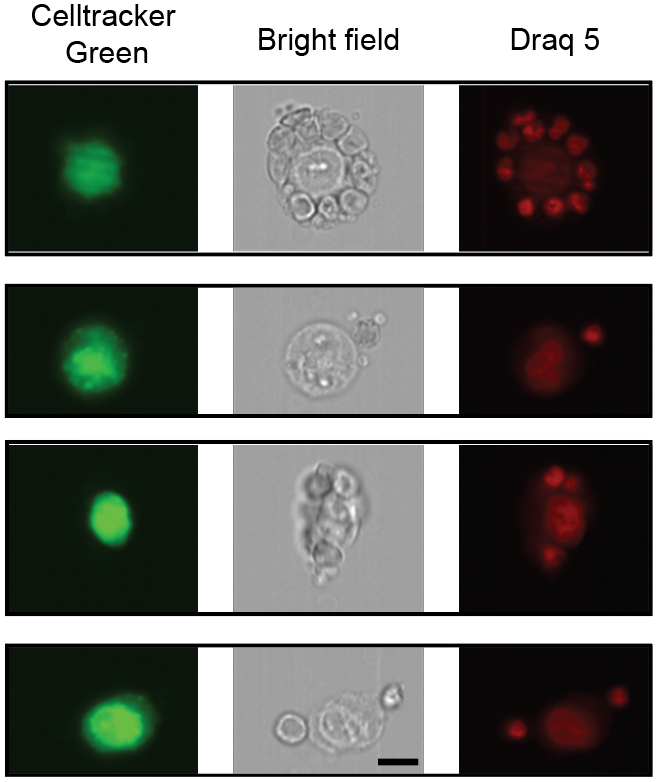


**Fig. S5.** Images of recovered HeLa cells attached with white blood cell(s) after the 2-step sorting illustrated in Fig.4. Specifically, HeLa cells were labelled with Celltracker Green (left panel) before being spiked into whole blood. After sorting, Draq 5 was added to the product to stain the cell nucleus of HeLa cells and WBCs (right panel). Images were acquired using imaging flow cytometry. Scale bar is 10 µm.


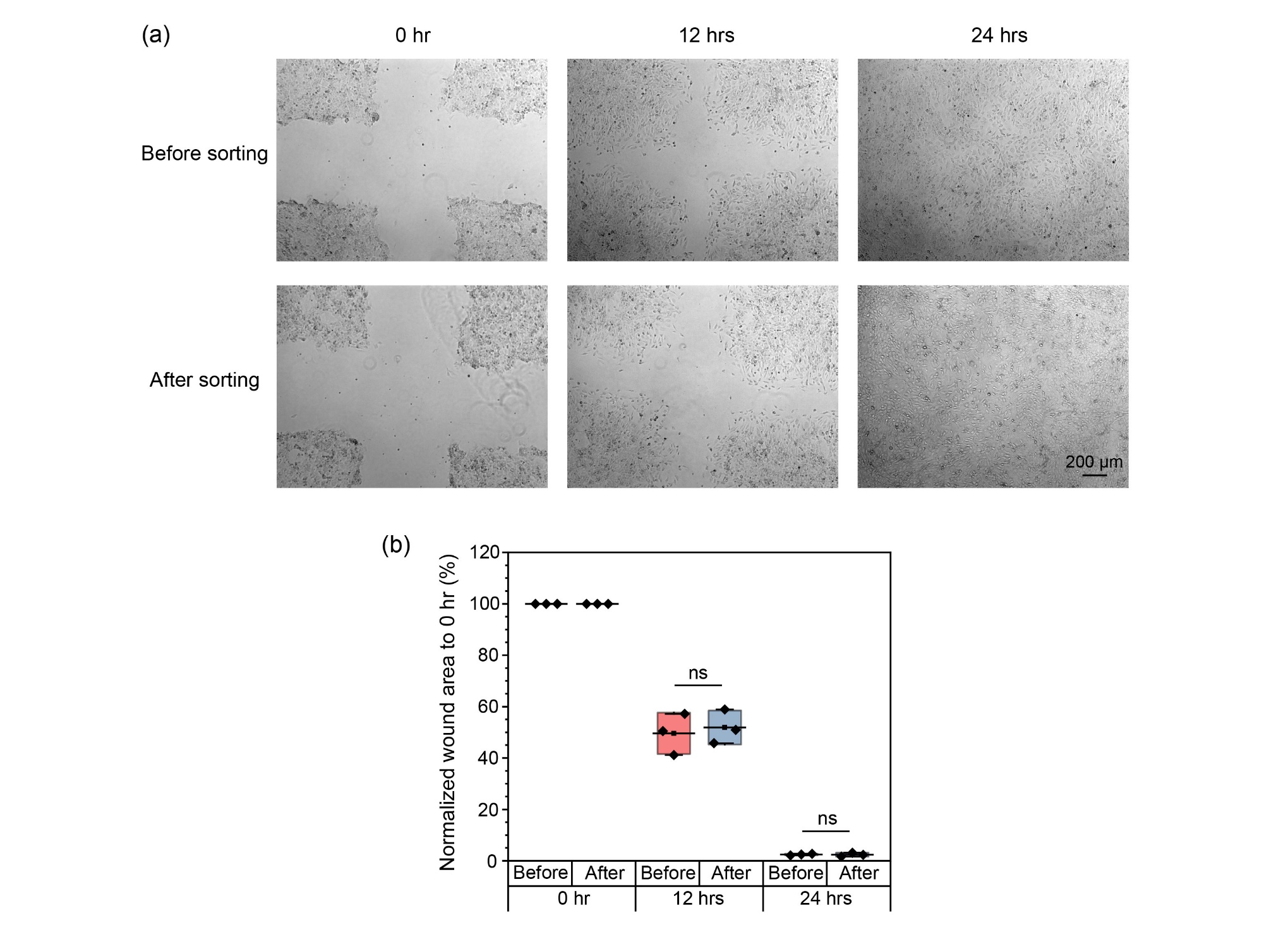


**Fig. S6**. Assessment of cell migration capacity of HeLa cells before vs. after microfluidic sorting. **(a)** Scratch assay was performed by first creating wound areas in cellular monolayer, then measuring normalized wound area to 0 hr during subsequent culture. Representative images of the wound area at 0, 12, and 24 hrs were shown. **(b)** Comparison of normalized wound area using HeLa cells before and after sorting (n=3). A paired two-tailed t-test was used for statistical analysis, ns, p>0.05.


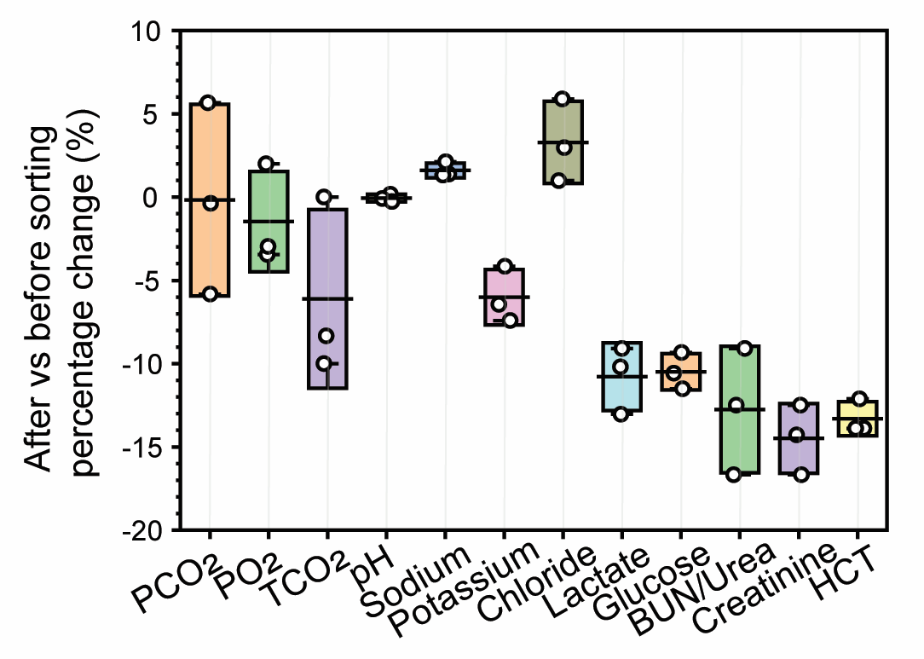


**Fig. S7**. After microfluidic sorting, relative percentage change of blood chemistry was measured by CHEM8+ and CG4+ cartridges. Note that the results may be skewed by the accuracy (i.e., significant digits) of the point-of-care cartridges and reader (Abbott), especially for analytes with low concentrations such as lactate and creatinine.


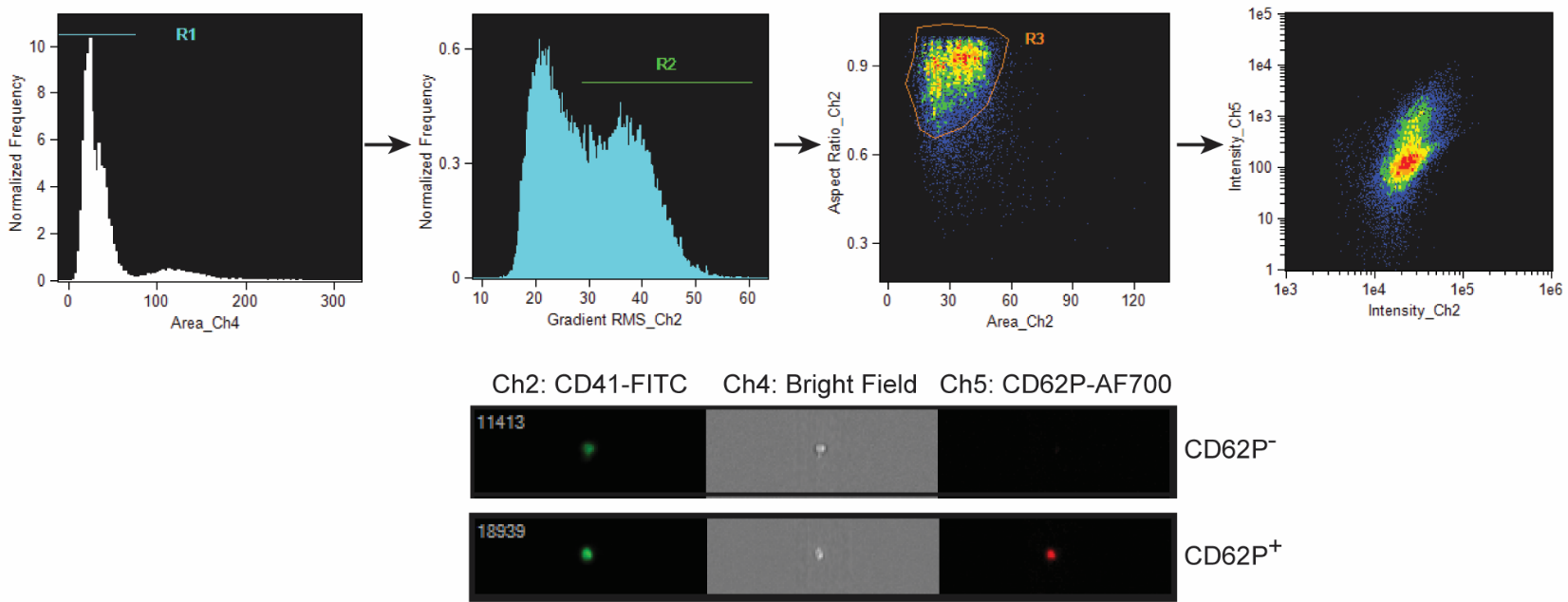


**Fig. S8.** Gating strategy for evaluation of platelet activation using imaging flow cytometry.


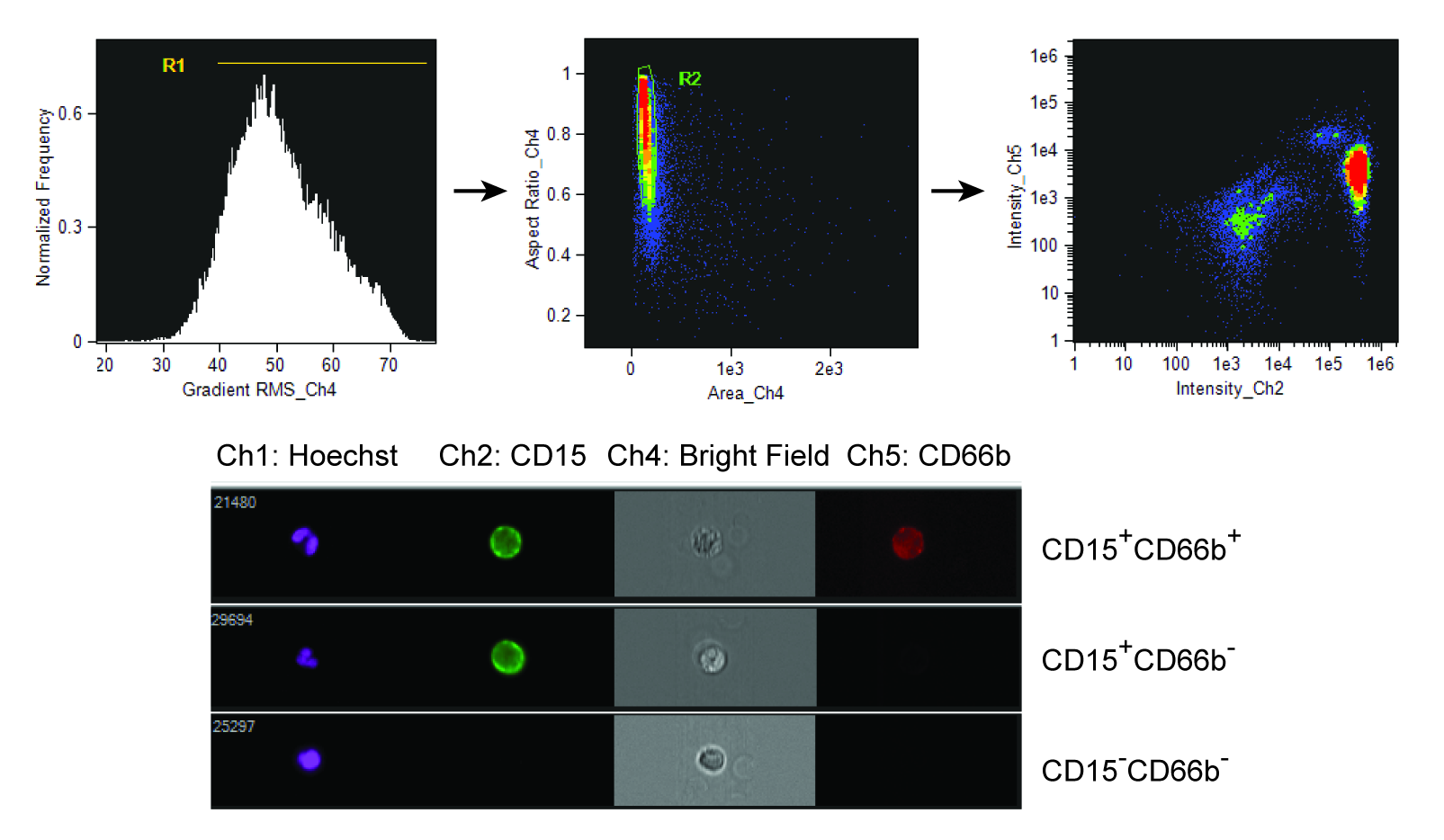


**Fig. S9.** Gating strategy for evaluation of neutrophil activation using imaging flow cytometry.


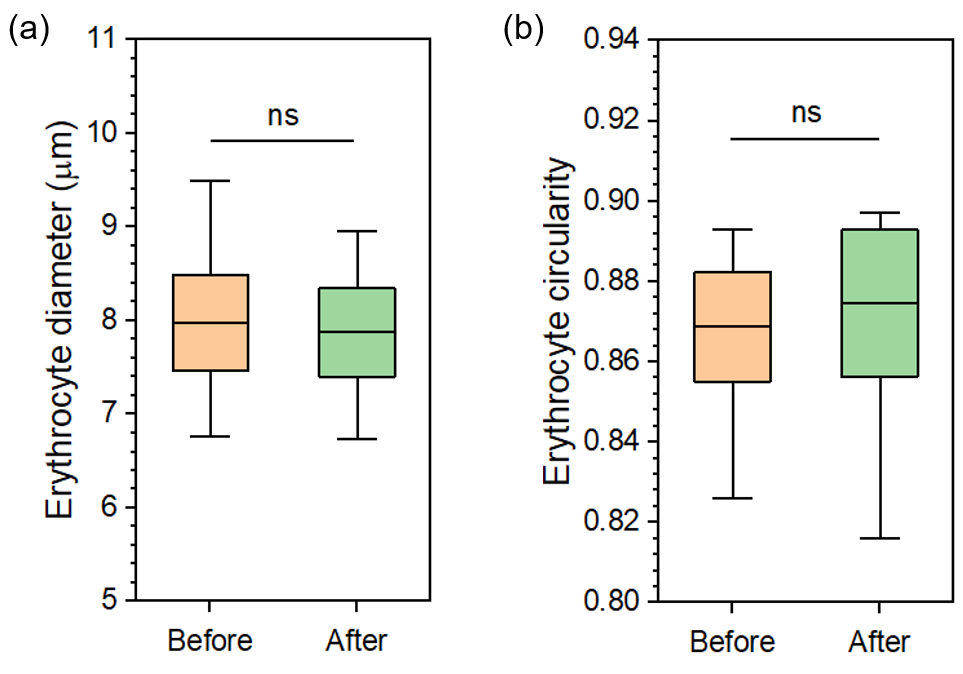


**Fig. S10.** Comparison of erythrocyte diameter and circularity before vs after microfluidic sorting. Circularity is calculated as 4π*(area/perimeter^2^). 1000 erythrocytes were analyzed, and two-tailed paired t-test was used for statistical analysis. ns, p> 0.05. Box and horizontal line represent standard deviation and mean respectively, whiskers represent max and min.


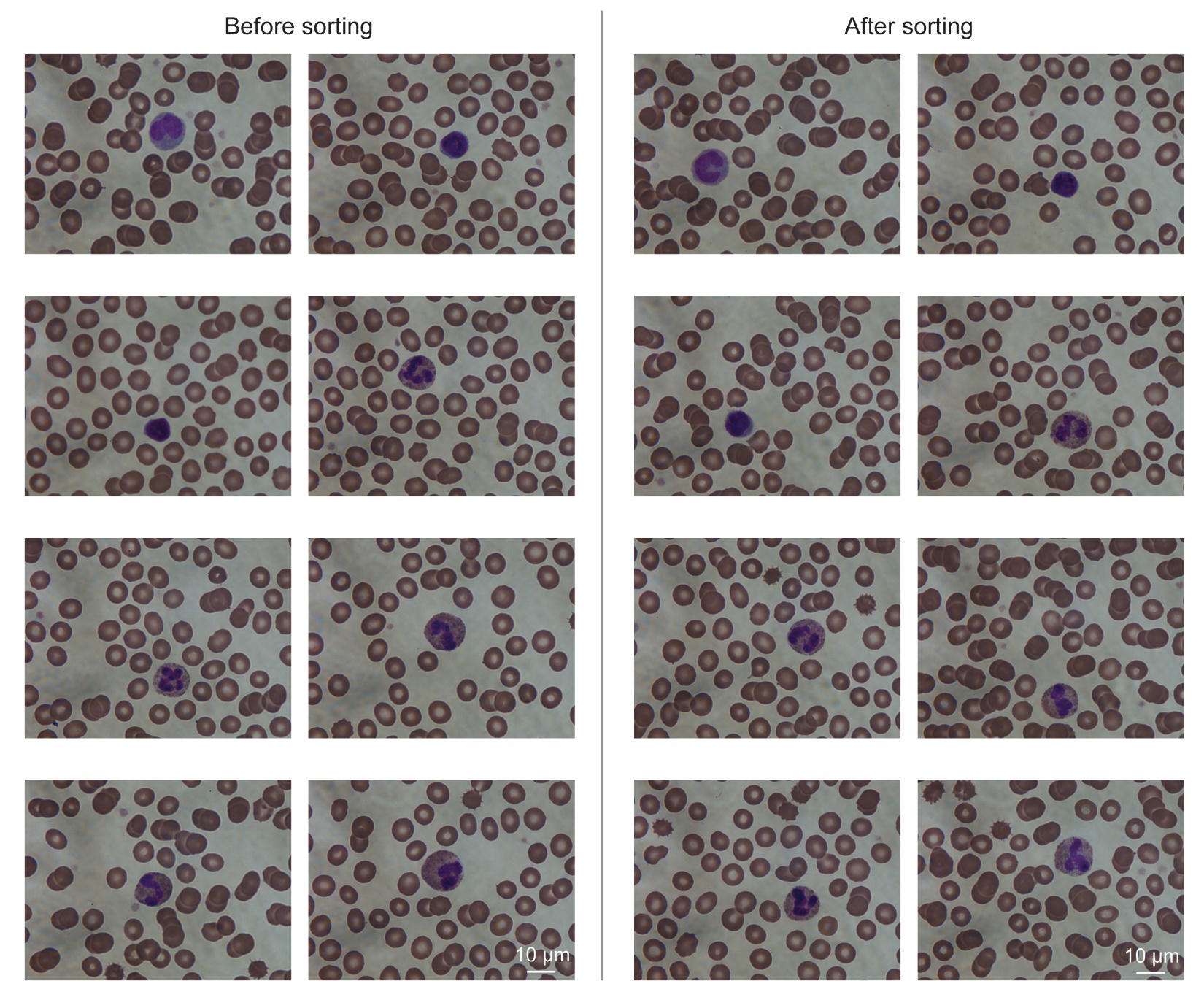


**Fig. S11.** Wright-Giemsa staining of blood smears before and after microfluidic sorting.

**References**

1 Boya, M. *et al.* High throughput, label-free isolation of circulating tumor cell clusters in meshed microwells. *Nature communications* **13**, 3385 (2022).

2 Miller, M. C., Robinson, P. S., Wagner, C. & O'Shannessy, D. J. The Parsortix™ cell separation system—A versatile liquid biopsy platform. *Cytometry Part A* **93**, 1234-1239 (2018).

3 Sarioglu, A. F. *et al.* A microfluidic device for label-free, physical capture of circulating tumor cell clusters. *Nature methods* **12**, 685-691 (2015).

4 Green, B. J. *et al.* PillarX: a microfluidic device to profile circulating tumor cell clusters based on geometry, deformability, and epithelial state. *Small* **18**, 2106097 (2022).

5 Kim, T. H. *et al.* A temporary indwelling intravascular aphaeretic system for in vivo enrichment of circulating tumor cells. *Nature communications* **10**, 1478 (2019).

6 Stott, S. L. *et al.* Isolation of circulating tumor cells using a microvortex-generating herringbone-chip. *Proceedings of the National Academy of Sciences* **107**, 18392-18397 (2010).

7 Au, S. H. *et al.* Microfluidic isolation of circulating tumor cell clusters by size and asymmetry. *Scientific reports* **7**, 2433 (2017).

8 Loutherback, K. *et al.* Deterministic separation of cancer cells from blood at 10 mL/min. *AIP advances* **2** (2012).

9 Edd, J. F. *et al.* Microfluidic concentration and separation of circulating tumor cell clusters from large blood volumes. *Lab on a Chip* **20**, 558-567 (2020).

10 Sollier, E. *et al.* Size-selective collection of circulating tumor cells using Vortex technology. *Lab on a Chip* **14**, 63-77 (2014).
